# Structural Characterization and Thermoelectric Properties of Br-Doped AgSn*_m_*[Sb_0.8_Bi_0.2_]Te_2+*m*_ Systems

**DOI:** 10.3390/ma16155213

**Published:** 2023-07-25

**Authors:** Daniela Delgado, Silvana Moris, Paulina Valencia-Gálvez, María Luisa López, Inmaculada Álvarez-Serrano, Graeme R. Blake, Antonio Galdámez

**Affiliations:** 1 Departamento de Química, Facultad de Ciencias, Universidad de Chile, Las Palmeras 3425, Santiago 7800003, Chile; daniela.delgado@ug.uchile.cl (D.D.); paulygav@uchile.cl (P.V.-G.); 2 Centro de Investigación de Estudios Avanzados del Maule (CIEAM), Vicerrectoría de Investigación y Postgrado, Universidad Católica del Maule, Avenida San Miguel 3605, Talca 3480112, Chile; smoris@ucm.cl; 3 Departamento de Química Inorgánica, Facultad de Ciencias Químicas, Universidad Complutense, 28040 Madrid, Spain; marisal@quim.ucm.es (M.L.L.); ias@quim.ucm.es (I.Á.-S.); 4 Zernike Institute for Advanced Materials, University of Groningen, Nijenborgh 4, 9747 AG Groningen, The Netherlands

**Keywords:** tellurides, Seebeck coefficient, thermal conductivity, lead-free thermoelectric materials

## Abstract

Herein, we report the synthesis, structural and microstructural characterization, and thermoelectric properties of AgSn*_m_*[Sb_0.8_Bi_0.2_]Te_2+*m*_ and Br-doped telluride systems. These compounds were prepared by solid-state reaction at high temperature. Powder X-ray diffraction data reveal that these samples exhibit crystal structures related to the NaCl-type lattice. The microstructures and morphologies are investigated by scanning electron microscopy, energy-dispersive X-ray spectroscopy (EDS), and high-resolution transmission electron microscopy (HRTEM). Positive values of the Seebeck coefficient (S) indicate that the transport properties are dominated by holes. The S of undoped AgSn*_m_*[Sb_0.8_Bi_0.2_]Te_2+*m*_ ranges from +40 to 57 μV·K^−1^. Br-doped samples with *m* = 2 show S values of +74 μV·K^−1^ at RT, and the Seebeck coefficient increases almost linearly with increasing temperature. The total thermal conductivity (*κ*_tot_) monotonically increases with increasing temperature (10–300 K). The *κ*_tot_ values of undoped AgSn*_m_*[Sb_0.8_Bi_0.2_]Te_2+*m*_ are ~1.8 W m^−1 ^K^−1^ (*m* = 4) and ~1.0 W m^−1^ K^−1^ (*m* = 2) at 300 K. The electrical conductivity (*σ*) decreases almost linearly with increasing temperature, indicating metal-like behavior. The ZT value increases as a function of temperature. A maximum ZT value of ~0.07 is achieved at room temperature for the Br-doped phase with *m* = 4.

## 1. Introduction

In recent years, the challenge of finding new sources of renewable energy that can generate power from waste heat has attracted considerable interest. It has been estimated that only one-third of produced energy is used efficiently, while the remaining two-thirds are discarded, mainly as waste heat. Therefore, taking advantage of this form of energy would result in an increase in energy efficiency [1,2]. Thermoelectric materials can be used for this purpose due to their ability to generate a potential difference (ΔV) from a temperature gradient (ΔT). The efficiency of these materials is determined by the dimensionless figure of merit (ZT) defined as ZT = (σS^2^/*κ*_tot_)T, where T is the temperature, S is the Seebeck coefficient, σ is the electrical conductivity, σS^2^ is the thermopower, and *κ* is the thermal conductivity given by *κ*_tot_ = *κ*_e_+ *κ*_latt_ (electronic thermal conductivity and lattice thermal conductivity, respectively) [3,4,5,6,7,8].

One of the most common and well-studied thermoelectric materials is PbTe due to its high efficiency; however, the presence of Pb has limited its applications. There has been interest in SnTe as a similar alternative, but spontaneously formed Sn vacancies induce a high carrier concentration, which leads to a low Seebeck coefficient and a high electric contribution to the thermal conductivity [9,10,11]. To increase the efficiency of this material by decreasing the carrier concentration, alloys with AgSbTe_2_ and AgBiTe_2_ have been previously reported, which form quaternary compounds with the general formula AgSn*_m_*MTe*_m_*_+2_ (M = Sb, Bi) [12,13,14,15,16].

To enhance the efficiency of materials that contain Sb in their composition, different types of doping have been reported in recent years, including the use of Bi to replace Sb. Bi has an atomic weight greater than that of Sb, which can increase phonon dispersion, leading to a decrease in the lattice thermal conductivity and, therefore, the total thermal conductivity [15,16]. In 2013, Mohanraman et al. [17] studied the effect of this substitution on *p*-type Ag(Sb_1−*x*_Bi*_x_*)Te_2_ material (*x* = 0; 0.03; 0.05; 0.07; 0.1) and reported a decrease in *κ*_latt_, with a minimum value of 0.38 Wm^−1^ K^−1^ for *x* = 0.05 at 510 K compared to the value obtained for AgSbTe_2_ (0.52 Wm^−1^ K^−1^). In 2015, Guin et al. [18] obtained an increase in electrical conductivity from 5 to 51 S cm^−1^ when AgSnSe_2_ was doped with 2% Bi, which led to an increase in ZT compared to the pristine sample. Tan et al. studied the effects of replacing all Sb in AgSn*_m_*SbTe*_m_*_+2_ with Bi and reported that Bi is more efficient at neutralizing the Sn vacancies in SnTe than Sb, which leads to a higher Seebeck coefficient [15].

On the other hand, studies with the aim of improving the thermoelectric properties of PbTe have reported the use of halogen anions such as Cl^−^, I^−^, or Br^−^. In 2011, Lalonde et al. reported an improvement in the electrical properties of PbTe when doped with iodine (*n*-type) at a temperatures between 700 and 800 K, obtaining a decrease in resistivity [19]. In 2018, Li et al. reported a study of SnSe doped with bromine, which caused a fourfold increase in the thermopower for the composition SnSe_0.9_Br_0.147_ compared to the pristine sample (4.5 W cm^−1^ K^−2^ and 1.1 W cm^−1^ K^−2^, respectively) [20]. In addition, doping generates a change in semiconductor behavior, from *p*-type to *n*-type, which is reflected by Hall-effect measurements. The ZT values obtained for samples doped with Br were larger than those for polycrystalline SnSe and can be compared to the values obtained for SnSe single crystals [20]. Guin et al. reported improvements in the room-temperature electrical conductivity of AgBiSe_2_ (*n*-type) by doping with chlorine, bromine, or iodine. This increase was mainly due to an increase in the carrier concentration, from 5.85 × 10^18^ (AgBiSe_2_) to 3.72 × 10^19^ carriers per cm^3^ (AgBiSe_1.98_Cl_0.02_), while the mobility decreased slightly for samples doped with 2% halogen compared to the pristine material [18].

In this work, we report the structural characterization and thermoelectric properties of lead-free systems with the general formula of AgSn*_m_*[Sb_0.8_Bi_0.2_]Te_2+*m*_ and doped with Br (*m* = 2 and 4). These compounds were synthesized at 1223 K using solid-state reactions. Powder X-ray diffraction patterns fitted using the Rietveld method are consistent with phases related to the cubic NaCl-type lattice. Scanning electron microscopy (SEM) and high-resolution transmission electron microscopy (HRTEM) were used to investigate the microstructures and morphologies of these systems. The electrical and thermal transport properties of the samples at low temperature were characterized by measurements of the Seebeck effect, thermal conductivity, and electrical conductivity. The figure of merit (ZT) for temperatures from 10 K to 300 K was evaluated.

## 2. Experimental Methods

AgSn*_m_*[Sb_0.8_Bi_0.2_]Te_2+*m*_ and Br-doped samples were synthesized under a dry and oxygen-free argon atmosphere using silver powder (99.99% purity, Aldrich, Saint Louis, MO, USA), antimony powder, (99.99% purity, Aldrich), tin powder (99.9% purity, Aldrich), tellurium powder (99.99% purity, Aldrich), bismuth powder (99.99% purity, Aldrich), and tin (II) bromide (Aldrich). Phases with the nominal compositions listed above were prepared via the solid-state reaction of powders of Ag, Sn, Bi, Sb, and Te (as well as SnBr_2_ for the Br-doped phases) mixed in stoichiometric proportions, then placed inside evacuated quartz ampoules. The reaction mixture was gradually heated to 1223 K at a rate of +423 K/h and maintained at this temperature for ~16 h. Then, the furnace was cooled to room temperature. The AgSn*_m_*[Sb_0.8_Bi_0.2_]Te_2+*m*_ (*m* = 2, 4,) system shows congruent melting points at ~680 and ~720 °C. A comparison of the PXRD patterns before and after DSC/TG analyses showed no significant changes. The melting point decreased from 700 °C to 680 °C when Bi was substituted by Sb in the pristine phase (*m* = 4). The same trend was shown when doped with bromine, where the melting point decreased from 720 °C to 660 °C (*m* = 4). For electrical measurements, the samples obtained via the solid-state reactions were crushed into powders and placed into a quartz cell with a parallelepiped shape. This quartz cell was placed into a Schlenk tube under an argon atmosphere to prevent oxidation of the bulk by air. This tube was then placed into a furnace at 1223 K for 20 min and quickly removed. The obtained ingots were cut and polished for measurements of their electrical transport and thermal properties, with approximate dimensions of 3 × 3 × 8 mm^3^. The density was calculated from the sample’s geometry and mass. Table S1 (Supplementary Materials) shows the percentage density of the parallelepiped-shaped samples (>92% theoretical density for all samples).

XRD patterns were obtained at RT using a Bruker D8 Advance powder diffractometer (Bruker, Billerica, MA, USA) with CuKα radiation over the 2θ range of 5°–80° at a step size of 0.01. The collected data were analyzed by Fullprof Rietveld refinement software (https://www.ill.eu/sites/fullprof/php/downloads.html) [21]. A standard LaB_6_ sample was used to determine the instrumental profiles. The chemical compositions of the samples were determined by scanning electron microscopy (SEM, JEOL 5400 system, Tokyo, Japan) and energy-dispersive X-ray spectroscopy (EDS, Oxford LinK ISIS microanalyzer, Oxford Instruments, Abingdon, UK). High-resolution transmission electron microscopy (HRTEM) and electron diffraction (ED) patterns were obtained using a JEOL JEM 2100 operating at an accelerating voltage of 300 kV. Samples were prepared by crushing the powders under n-butanol and dispersing them over copper grids covered with a porous carbon film. Semiquantitative chemical analyses were carried out using EDS. Differential scanning calorimetry (DSC) and thermogravimetric analysis (TG) were performed on a Rheometric Scientific STA 1500H/625 thermal analysis system. DSC/TG curves were acquired simultaneously for each sample over a temperature range from room temperature to 1273 K; the samples were heated at 10 K min^−1^ under flowing argon. Low-temperature thermoelectric properties were obtained in a helium-cooled cryostat using a PPMS system (Quantum Design) for temperatures from 10 K to 300 K. Hall-effect measurements were performed using an ECOPIA HMS 2000 system. Electrical contacts of gold were deposited by sputtering on pellets used in the thermoelectric measurements. The Hall coefficient at a field of ±0.556 T was obtained from linear fits of the Hall resistivity using the van der Pauw method at RT.

## 3. Results and Discussion

### 3.1. X-ray Powder Diffraction (XRD) and Electron Microscopic Characterization (SEM-EDS and HRTEM)

**Figure 1:**
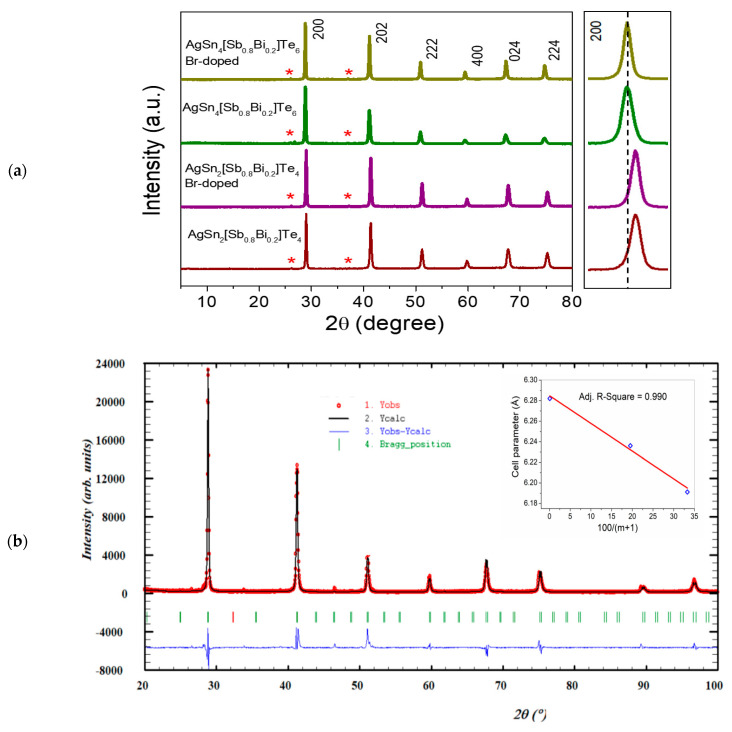
(**a**) XRD patterns and *hkl* peak indexing based on the cubic unit cell for AgSn*_m_*[Sb_0.8_Bi_0.2_]Te_2+*m*_ systems at room temperature; the enlarged pattern shows the 200 peak in the range of 26° to 31° in 2θ_._ The red asterisks indicate reflections associated with an unidentified impurity. (**b**) Observed, calculated, and difference XRD profiles of AgSn_4_[Sb_0.8_Bi_0.2_]Te_6_ fitted using the Rietveld method with Fullprof software (https://www.ill.eu/sites/fullprof/php/downloads.html). The inset shows a plot of the *a*-lattice parameter as a function of the molar ratio (100/(1 + *m*)).

The XRD patterns of all samples were fully indexed in the *Pm-3m* space group, with the exception of two very weak impurity peaks, within the detection limits of the technique. The shape and intensity of the XRD peaks indicate the high crystallinity of all the telluride samples, as shown in Figure 1. The experimental XRD patterns were compared with those of previously reported pristine AgSn*_m_*SbTe_2+*m*_ samples, indicating that they are isostructural, and the measured *d*-spacings are in good agreement with the calculated values. Increasing Sn content in AgSn*_m_*[Sb_0.8_Bi_0.2_]Te_2+*m*_ leads to an increase in the cell parameters (Table S1, Supplementary Materials). For the Br-doped compounds, the cell parameters increase gradually as tellurium is replaced by bromine. Figure 1b displays the Rietveld refinement profile at room temperature for AgSn_4_[Sb_0.8_Bi_0.2_]Te_6_ using the cubic model. The *R* indices obtained by Rietveld refinement are shown in Table S1. Similar structures have previously been reported for AgSn[Bi_1*−x*_Sb*_x_*]Se_3_ samples [22]. The experimental results provide evidence that the AgSn*_m_*[Sb_0.8_Bi_0.2_]Te_2+*m*_ and Br-doped compounds have a cubic NaCl-type lattice.

The inset in Figure 1b shows the *a*-lattice parameter as a function of Ag[Sb_0.8_Bi_0.2_]Te_2_% plotted in the form of the 100/(1 + *m*) molar ratio. This linear dependence has previously been observed for AgSn*_x_*BiTe*_x_*_+2_ and AgSn*_m_*SbSe_2_Te*_m_* systems [15,23].

**Figure 2:**
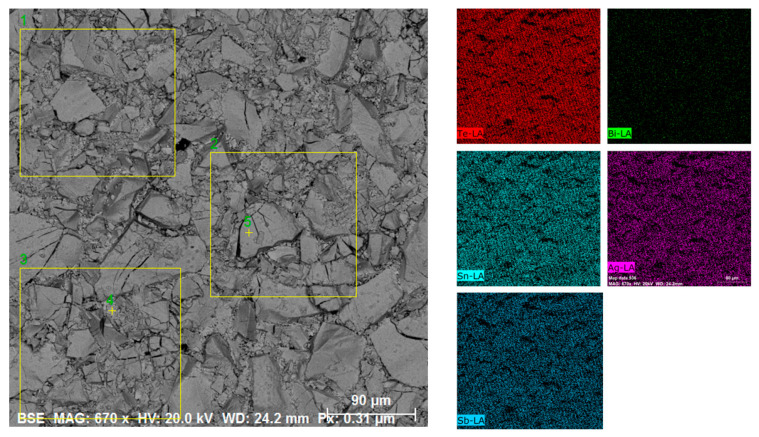
Representative SEM-EDS analysis of AgSn_2_[Sb_0.8_Bi_0.2_]Te_4_: backscattered electron (BSE) and energy-dispersive X-ray (EDS) chemical mapping images from powder sample.

SEM-EDS analyses of the powder samples indicate that the chemical composition of all the phases is uniform throughout the scanned region, within the detection limits of the technique, as represented for AgSn_2_[Sb_0.8_Bi_0.2_]Te_4_ (Figure 2 and Table 1) and AgSn_4_[Sb_0.8_Bi_0.2_]Te_5.97_Br_0.03_ (Figure S1, Supplementary Materials). EDS microprobe chemical analysis performed on several areas of the AgSn_2_[Sb_0.8_Bi_0.2_]Te_4_ sample revealed an average chemical composition consistent with the nominal composition (as shown in Figure 2, yellow squares). The chemical distributions of bismuth, antimony, silver, tellurium, tin, and bromine in both samples are homogeneous.

**Figure 3:**
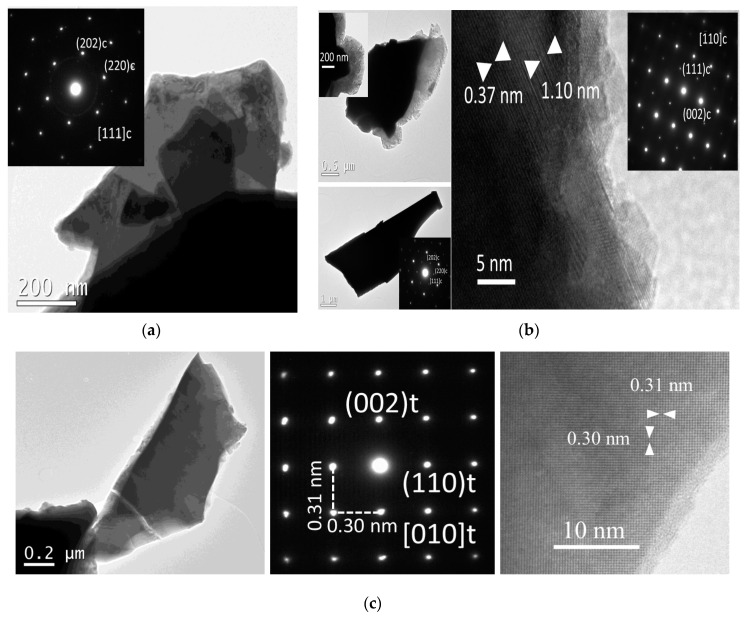
HRTEM images and ED patterns of different selected regions of AgSn_2_[Sb_0.8_Bi_0.2_]Te_4_ (**a**), AgSn_4_[Sb_0.8_Bi_0.2_]Te_6_ (**b**), and AgSn_2_Sb_0.8_Bi_0.2_Te_3.97_Br_0.03_ (**c**). The high-magnification images and ED patterns in (**a**) and (**b**) are coherent, with cubic symmetry along zone axes [111]*_c_* and [110]*_c_*. The images exhibit regions with intergrowths and local deviation from cubic symmetry. The images and ED pattern in (**c**) show crystals of micrometer size that have local regions with tetragonal symmetry.

HRTEM analysis and electron diffraction (ED) patterns show the microstructural characteristics of the telluride samples. Small crystals of between 1 and 5 µm with irregular shapes are observed. ED patterns obtained along the [111]*c* and [110]*c* zone axes for different selected regions of AgSn_2_[Sb_0.8_Bi_0.2_]Te_4_ and AgSn_4_[Sb_0.8_Bi_0.2_]Te_6_ are shown in Figure 3a,b.

Electron diffraction (ED) patterns and distances associated with lattice fringes in the HRTEM images of AgSn*_m_*[Sb_0.8_Bi_0.2_]Te_2+*m*_ and the Br-doped family of compounds are generally consistent with cubic symmetry. Figure 3c displays ED patterns obtained along the [111]*_c_* zone axis for different selected regions of AgSn_2_Sb_0.8_Bi_0.2_Te_3.97_Br_0.03_. Although most of the ED patterns are coherent with cubic symmetry, the separation between spots in the ED patterns for some crystallites suggests the existence of tetragonal regions. Some high-magnification images show nanoregions, intergrowths, and regions with very low crystallinity (see Figure 3b,c). Previously, Quarez et al. experimentally demonstrated that the cubic space group (*Pm-3m*) and a lower symmetric space group such as *P4/mmm* coexist in AgPb_m_SbTe_2+*m*_ phases by performing HRTEM analyses and single-crystal XRD [24].

The mean atomic compositions of AgSn_2_[Sb_0.8_Bi_0.2_]Te_4_ and AgSn_4_[Sb_0.8_Bi_0.2_]Te_6_ determined according EDS mapping data collected from the entire crystals are consistent with the nominal compositions within the detection limits of the technique (Tables S2–S4, Supplementary Materials). In the Br-doped phases, a homogeneous bromine distribution is observed in several crystals when measured in different regions. These EDS results from HRTEM are in agreement with the SEM-EDS analysis.

### 3.2. Thermoelectric Properties

**Figure 4:**
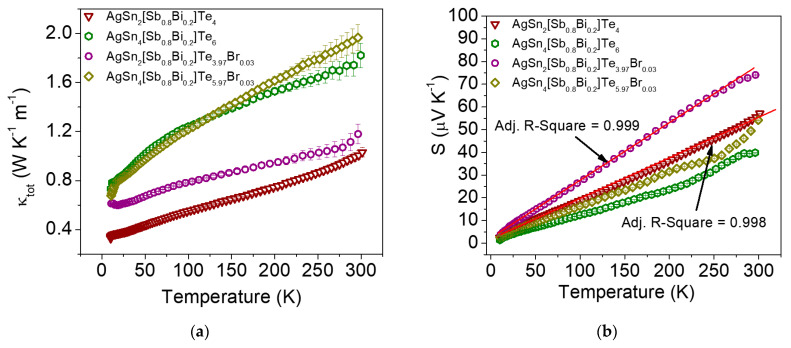
Temperature dependence of (**a**) total thermal conductivity *κ*_tot_ and (**b**) Seebeck coefficient S as a function of temperature for AgSn*_m_*[Sb_0.8_Bi_0.2_]Te_2+*m*_ and Br-doped phases. The solid red lines show representative linear fits to the experimental data in the low-temperature range.

The temperature dependence of the total thermal conductivity (*κ*_tot_) at low temperatures for the AgSn*_m_*[Sb_0.8_Bi_0.2_]Te_2+*m*_ and Br-doped phases is shown in Figure 4a. The thermal conductivity monotonically increases with increasing temperature. The *κ*_tot_ values are ~1.8 W m^−1 ^K^−1^ (*m* = 4) and ~1.0 W m^−1^ K^−1^ (*m* = 2) at 300 K. The AgSn*_m_*SbTe_2+*m*_ pristine phases have very low total thermal conductivity, ranging from ~1.5 to ~3.0 W m^−1^ K^−1^ at 300 K [13]. The *κ*_tot_ value of AgSn*_m_*[Sb_0.8_Bi_0.2_]Te_2+*m*_ (*m* = 2 in this work) is lower than the value reported for AgSn_5_SbTe_7_ (*κ*_tot_ = ~1.6 W m^−1 ^K^−1^ at RT) [16]. The partial chemical substitution of 20 mol% bismuth by antimony can therefore decrease the *κ*_tot_ of AgSn*_m_*SbTe_2+*m*_. The Br-doped phases show similar values of *κ*_tot_ throughout the studied temperature range. For example, the *κ*_tot_ of AgSn_4_[Sb_0.8_Bi_0.2_]Te_5.97_Br_0.03_ is ~2.0 W m^−1 ^K^−1^ and ~1.1 W m^−1 ^K^−1^ at 300 and 70 K, respectively. A lower *κ*_tot_ is obtained for AgSn_2_[Sb_0.8_Bi_0.2_]Te_3.97_Br_0.03_ (~1.2 W m^−1 ^K^−1^ at RT).

Figure 4b shows the temperature dependence of the Seebeck coefficient (S) of the AgSn*_m_*[Sb_1*−x*_Bi*_x_*]Te_2+*m*_ (*x* = 0.2; *m* = 2 and 4) and Br-doped samples. An almost linear increase in the Seebeck coefficient is observed between 10 and 300 K. These results were checked by performing several heating–cooling cycles on each sample. The S values of the AgSn*_m_*[Sb_0.8_Bi_0.2_]Te_2+*m*_ systems (*m* = 2 and 4) obtained via the isoelectronic substitution of a fraction of Sb atoms with Bi indicate that holes are the dominant conduction carriers.

The Seebeck coefficient of AgSn*_m_*[Sb_0.8_Bi_0.2_]Te_2+*m*_ increases from +40 μV·K^−1^ (*m* = 4) to +57 μV·K^−1^ (*m* = 2) with decreasing Sn content at 300 K. This value is approximately 1.5 times lower than that of Ag_0.8_SnSb_1.15_Te_3_ (~+80 μV·K^−1^) and is comparable with the Seebeck coefficient of SnTe at 300 K (+40 μV·K^−1^) and the S values of ~+80 to 30 μV·K^−1^ for SnTe-AgSbTe_2_ systems at RT [16,25,26]. Han et al. reported that the Seebeck coefficients of *p*-type AgSn*_m_*SbTe_2+*m*_ decreased from ~+75 μV·K^−1^ for *m* = 10 to ~+50 μV·K^−1^ for *m* = 2 at RT [13]. In the systems measured in the current study, the chemical substitution of Bi for Sb does not considerably increase the S values compared to the pristine phases. However, bromine doping contributes to enhancement of the Seebeck coefficient. The highest *S* value obtained is +74 μV·K^−1^ for AgSn_2_[Sb_0.8_Bi_0.2_]Te_3.97_Br_0.03_ compared to +57 μV·K^−1^ for the non-doped phase (*m* = 2). This value is comparable to the Seebeck coefficient of the Ag_0.8_SnSb_1.15_Te_3_ and SnTe-AgBiTe_2_ systems at RT [16,25,26]. Doping with Br increases the S coefficient from +40 μV·K^−1^ to +57 μV·K^−1^ for *m* = 4, AgSn_4_[Sb_0.8_Bi_0.2_]Te_5.97_Br_0.03_, but this increase is considerably smaller than that observed for *m* = 2 at RT.

The temperature dependence of the Seebeck coefficient typical of metallic or degenerate semiconductors is expressed by the following formula:

(1) $$S=\left[\frac{8\pi^{2/3}k_{B}^{2}\left(r+3/2\right)}{3^{5/3}eh^{2}}\right]\left(\frac{m^{*}}{n^{2/3}}\right)T$$

where *S* is the Seebeck coefficient, m* is the effective mass, *k_B_* is Boltzmann’s constant, *e* is the charge of an electron, *h* is Planck’s constant, and *n* is the carrier concentration [3,7]. The Seebeck coefficient was fitted in the temperature range from 10 K to 300 K for AgSn_2_[Sb_0.8_Bi_0.2_]Te_4_ and AgSn_2_[Sb_0.8_Bi_0.2_]Te_3.97_Br_0.03_, as shown in Figure 4b. Hall-effect measurements revealed that the carrier concentration of the telluride samples was in the range of +2.5–1.7 × 10^19^ cm^−3^ at room temperature. These measurements imply an m* of ~0.91·*m*_0_ assuming an acoustic phonon scattering mechanism (*r* = −1/2) and an m* of ~0.32·*m*_0_ assuming an ionized impurity scattering mechanism (*r* = 3/2). Density-of-states effective masses, m* ~ 0.61−0.99·*m*_0_ and m* ~ 0.32·*m*_0_ at room temperature, were previously reported for Sn_0.85_Sb_0.15_Te and PbTe, respectively [27,28].

**Figure 5:**
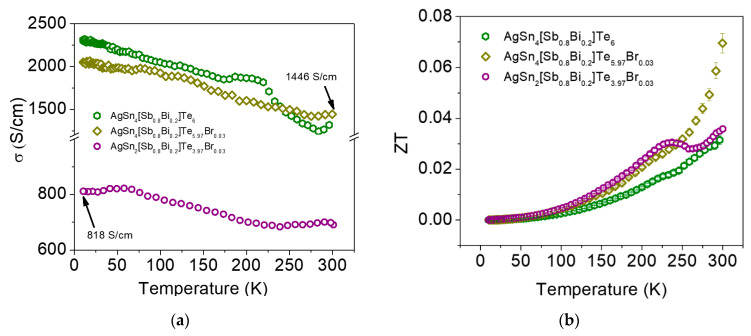
Temperature dependence of (**a**) the electrical conductivity (*σ*) and (**b**) figure of merit (*Z*T) for AgSn*_m_*[Sb_0.8_Bi_0.2_]Te_2+*m*_ and Br-doped phases.

Figure 5a shows plots of the temperature dependence of the electrical conductivity (*σ*) for AgSn_4_[Sb_0.8_Bi_0.2_]Te_6_ and the Br-doped phases. *σ* decreases almost linearly with increasing temperature, indicating that the samples show metal-like behavior. AgSn_4_[Sb_0.8_Bi_0.2_]Te_6_ has similar electrical conductivity to the Br-doped phases and lower values than the pristine phases (Table 2). For example, our measured value of *σ* is 1232 S cm^−1^ for AgSn*_m_*[Sb_0.8_Bi_0.2_]Te_2+*m*_ (*m* = 4), and the reported *σ* value of AgSn_2_SbTe_4_ is ~1800 S cm^−1^ at 300 K [13]. The figure of merit (*Z*T) is plotted as a function of temperature for AgSn_4_[Sb_0.8_Bi_0.2_]Te_6_ and the Br-doped samples in Figure 5b. The samples show monotonically increasing *Z*T with Br doping. A maximum *Z*T value of ~0.07 is achieved at room temperature for AgSn_4_[Sb_0.8_Bi_0.2_]Te_5.97_Br_0.03_.

## 4. Conclusions

In summary, polycrystalline AgSn*_m_*[Sb_0.8_Bi_0.2_]Te_2+*m*_ and Br-doped phases were successfully prepared at high temperatures using solid-state reactions. EDS mapping analysis using SEM and HRTEM indicates that the chemical substitution of bismuth and bromine in the pristine AgSn*_m_*SbTe_2+*m*_ samples is homogeneous within experimental error. HRTEM images and electron diffraction patterns reveal the existence of nanoregions with different orientations or symmetries, whether cubic or tetragonal. X-ray diffraction (XRD) and electron diffraction (ED) data are consistent with a cubic NaCl-type superstructure. The Seebeck coefficient (S) of AgSn*_m_*[Sb_0.8_Bi_0.2_]Te_2+*m*_ ranges from +40 to 74 μV·K^−1^. The total thermal conductivity (*κ*_tot_) is decreased by bromine doping (~1.2 W m^−1 ^K^−1^ at RT). S and *κ*_tot_ increase over the studied temperature range, whereas the electrical conductivity (σ) decreases with increasing temperature. It is worth mentioning that all samples show metal-like behavior. Finally, a maximum ZT value of ~0.07 was obtained at room temperature for the Br-doped AgSn_4_[Sb_0.8_Bi_0.2_]Te_6_ phase. This study deepens the understanding of rocksalt-type telluride phases and provides a new approach for optimizing TE performance by introducing chemical substitutions.

## Figures and Tables

**Table 1:** Chemical analysis of AgSn_2_[Sb_0.8_Bi_0.2_]Te_4_ nominal composition performed on several areas of the sample (See BSE-Figure 2). The chemical formula averaged over these areas is shown.

Spectrum	Mass Percentage (%)					Average Chemical Formula
	Ag	Sn	Sb	Bi	Te	
1	10.12	23.39	9.66	6.89	49.94	Ag_1.00_Sn_2.06_[Sb_0.88_Bi_0.32_]Te_4.14_
2	10.01	23.21	9.92	7.58	49.28	
3	10.33	22.77	9.94	7.13	49.82	
4	10.04	24.54	9.39	4.96	51.07	
5	10.59	21.66	11.81	5.32	50.63	
Mean value:	10.22	23.11	10.14	6.37	50.15	
Sigma:	0.24	1.04	0.96	1.16	0.70	
Sigma mean:	0.11	0.47	0.43	0.52	0.32	

**Table 2:** Room-temperature physical properties, including Seebeck coefficient (S), electrical conductivity (*σ*), and Hall-carrier concentration (*n*).

	S (μV·K^−1^)	*σ* (S cm^−1^)	*n*^§^ (cm^−3^)
AgSn_4_[Sb_0.8_Bi_0.2_]Te_6_	+40	1429	+1.71 × 10^19^
AgSn_4_[Sb_0.8_Bi_0.2_]Te_5.97_Br_0.03_	+57	1443	+5.51 × 10^19^
AgSn_2_[Sb_0.8_Bi_0.2_]Te_3.97_Br_0.03_	+74	685	+2.12 × 10^19^

**^§^** Hall-effect measurements using the van der Pauw method.

## Data Availability

Data are contained within the article.

## Supplementary Materials

The following supporting information can be downloaded at: https://www.mdpi.com/article/10.3390/ma16155213/s1, Table S1: Lattice parameters and *R* indices obtained from Rietveld refinement of PXRD patterns; Figure S1: Representative energy-dispersive X-ray (EDS) chemical mapping images of AgSn_4_[Sb_0.8_Bi_0.2_]Te_5.97_Br_0.03_ powder sample; Table S2: EDS chemical analysis from HRTEM measurements of AgSn_2_Sb_0.8_Bi_0.2_Te_4_ samples collected from different crystals and zones; Table S3: EDS chemical analysis from HRTEM measurements of AgSn_4_Sb_0.8_Bi_0.2_Te_6_ samples collected from different crystals and zones; Table S4: EDS chemical analysis from HRTEM measurements of AgSn_4_Sb_0.8_Bi_0.2_Te_5.97_Br_0.03_ samples collected from different crystals and zones.
