# Supplementary material for: Structural Characterization and Thermoelectric Properties of Br-Doped AgSnm[Sb0.8Bi0.2]Te2+m Systems
Source: Materials (Basel). 2023 Jul 25;16(15):5213. doi: 10.3390/ma16155213 (PMC10419848; doi:10.3390/ma16155213)

materials MDPI

Structural Characterization and Thermoelectric Properties of Br-Doped $AgSn_m[Sb_{0.8}Bi_{0.2}]Te_{2+m}$ Systems

Daniela Delgado [1], Silvana Moris [2], Paulina Valencia-Gálvez [1], María Luisa López [3], Inmaculada Álvarez-Serrano [3], Graeme R. Blake [4,*] and Antonio Galdámez [1,*]

[1] Departamento de Química, Facultad de Ciencias, Universidad de Chile, Las Palmeras 3425, Santiago 7800003, Chile; daniela.delgado@ug.uchile.cl (D.D.); paulygav@uchile.cl (P.V.-G.)
[2] Centro de Investigación de Estudios Avanzados del Maule (CIEAM), Vicerrectoría de Investigación y Postgrado, Universidad Católica del Maule, Avenida San Miguel 3605, Talca 3480112, Chile; smoris@ucm.cl
[3] Departamento de Química Inorgánica, Facultad de Ciencias Químicas, Universidad Complutense, 28040 Madrid, Spain; marisal@quim.ucm.es (M.L.L.); ias@quim.ucm.es (I.Á.-S.)
[4] Zernike Institute for Advanced Materials, University of Groningen, Nijenborgh 4, 9747 AG Groningen, The Netherlands
* Correspondence: g.r.blake@rug.nl (G.R.B.); agaldamez@uchile.cl (A.G.)

Supplementary Materials

Table S1. Lattice parameters and *R*-indices obtained from Rietveld refinement of PXRD patterns, and relative densities of samples used in thermoelectric measurements.

Composition	*a* (Å)	% Densification	Bragg R-factor	Rf-factor
$AgSn_2[Sb_{0.8}Bi_{0.2}]Te_4$	6.19012(7)	93.4	9.86	10.4
$AgSn_2[Sb_{0.8}Bi_{0.2}]Te_{3.97}Br_{0.03}$	6.20021(5)	94.6	8.36	8.52
$AgSn_4[Sb_{0.8}Bi_{0.2}]Te_6$	6.23730(2)	94.2	8.46	6.18
$AgSn_4[Sb_{0.8}Bi_{0.2}]Te_{5.97}Br_{0.03}$	6.24030(1)	93.2	11.4	9.91

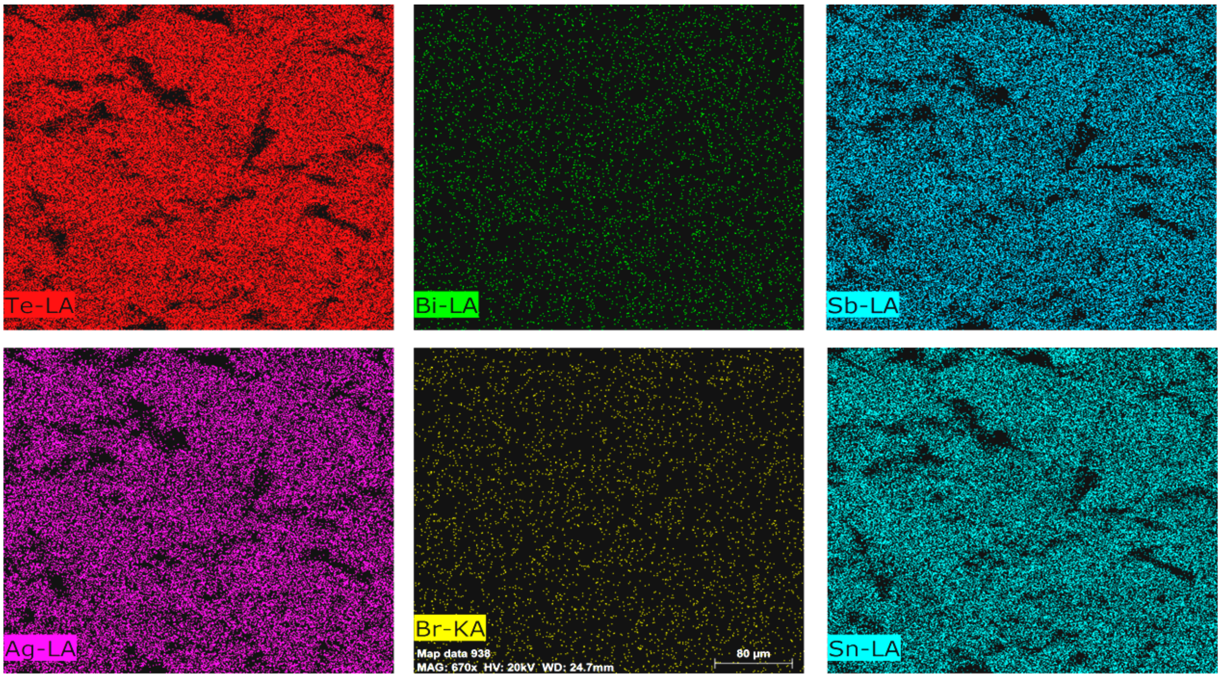

Figure S1. Representative energy dispersive x-ray (EDS) chemical mapping images of $AgSn_4[Sb_{0.8}Bi_{0.2}]Te_{5.97}Br_{0.03}$ powder sample.

Table S2. EDS chemical analysis from HRTEM measurements of samples with nominal composition $AgSn_2[Sb_{0.8}Bi_{0.2}]Te_4$ taken from different crystals and zones.

Crystal	Ag	Sn	Sb	Te	Bi	Chemical composition
1	11.10	29.33	4.44	52.00	3.13	$AgSn_{1.6}(Sb_{0.4}Bi_{0.3})Te_{4.7}$
2	12.88	22.67	9.01	51.80	3.64	$AgSn_{1.8}(Sb_{0.7}Bi_{0.3})Te_{4.0}$
3	13.91	21.99	8.77	52.24	3.09	$AgSn_{1.6}(Sb_{0.6}Bi_{0.2})Te_{3.8}$
4	14.69	20.85	10.40	50.74	3.33	$AgSn_{1.4}(Sb_{0.7}Bi_{0.2})Te_{3.5}$
5	14.16	23.81	8.22	50.43	3.38	$AgSn_{1.7}(Sb_{0.6}Bi_{0.2})Te_{3.6}$
Mean	13.17	22.71	8.30	51.43	4.39	$AgSn_{1.7}(Sb_{0.6}Bi_{0.3})Te_{3.9}$
Std. Deviation	1.48	3.24	1.86	0.67	2.31	

Table S3. EDS chemical analysis from HRTEM measurements of samples with nominal composition $AgSn_4[Sb_{0.8}Bi_{0.2}]Te_6$ taken from different crystals and zones.

Crystal	Ag	Sn	Sb	Te	Bi	Chemical composition
1a	7.60	34.53	4.12	51.83	1.91	$AgSn_{4.5}(Sb_{0.5}Bi_{0.3})Te_{6.8}$
1b	7.62	35.12	4.17	51.13	1.95	$AgSn_{4.6}(Sb_{0.5}Bi_{0.3})Te_{6.7}$
2	10.77	30.49	7.13	49.57	2.03	$AgSn_{2.8}(Sb_{0.7}Bi_{0.2})Te_{4.6}$
3a	6.92	35.54	4.45	51.19	1.90	$AgSn_{5.1}(Sb_{0.6}Bi_{0.3})Te_{7.4}$
3b	6.56	36.23	4.04	51.40	1.77	$AgSn_{5.5}(Sb_{0.6}Bi_{0.3})Te_{7.8}$
4	8.69	30.42	6.48	52.27	2.13	$AgSn_{3.5}(Sb_{0.7}Bi_{0.2})Te_{6.0}$
5a	8.61	29.20	7.41	52.14	2.65	$AgSn_{3.4}(Sb_{0.9}Bi_{0.3})Te_{6.1}$
5b	7.47	32.33	7.87	50.41	1.93	$AgSn_{4.3}(Sb_{1.1}Bi_{0.3})Te_{6.7}$
Mean	8.03	32.98	5.71	51.24	2.03	$AgSn_{4.1}(Sb_{0.7}Bi_{0.3})Te_{6.4}$
Std. Deviation	1.33	2.71	1.67	0.91	0.27	

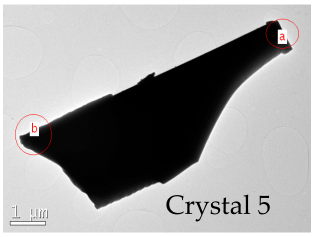

Table S4. EDS chemical analysis from HRTEM measurements of samples with nominal composition $AgSn_4[Sb_{0.8}Bi_{0.2}]Te_{5.97}Br_{0.03}$ taken from different crystals and zones.

Crystal	Br	Ag	Sn	Sb	Te	Bi
1	0.30	7.69	33.75	5.08	51.18	2.00
2a	0.29	8.28	32.75	4.64	51.94	2.08
2b	0.15	8.16	33.96	4.78	50.62	2.33
3	0.14	8.21	33.71	4.20	51.39	2.35
4a	0.38	8.45	32.32	5.13	51.19	2.53
4b	0.25	8.49	32.54	5.97	50.82	1.93
Mean	0.25	8.22	33.17	4.97	51.19	2.20
Std. Deviation	0.09	0.29	0.71	0.60	0.46	0.23

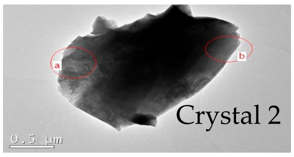

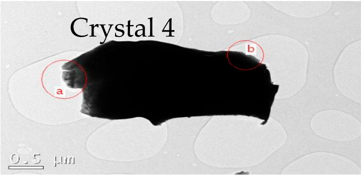

Supplement: Supplementary file 1 [file materials-16-05213-s001.zip › materials-2509847-supplementary.pdf]
